# Population Structure and Diversity in European Honey Bees (*Apis* *mellifera* L.)—An Empirical Comparison of Pool and Individual Whole-Genome Sequencing

**DOI:** 10.3390/genes13020182

**Published:** 2022-01-21

**Authors:** Chao Chen, Melanie Parejo, Jamal Momeni, Jorge Langa, Rasmus O. Nielsen, Wei Shi, SMARTBEES WP3 DIVERSITY CONTRIBUTORS, Rikke Vingborg, Per Kryger, Maria Bouga, Andone Estonba, Marina Meixner

**Affiliations:** 1 Institute of Apicultural Research, Chinese Academy of Agricultural Sciences, Beijing 100093, China; shiweibri@126.com; 2 Key Laboratory of Pollinating Insect Biology, Ministry of Agriculture and Rural Affairs, Beijing 100093, China; 3 Department of Genetics, Physical Anthropology and Animal Physiology, Faculty of Science and Technology, University of the Basque Country (UPV/EHU), 48940 Leioa, Spain; jorgeeliseo.langa@ehu.es (J.L.); andone.estonba@ehu.eus (A.E.); 4 Swiss Bee Research Center, Agroscope, 3003 Bern, Switzerland; 5 Eurofins Genomics, 8200 Aarhus, Denmark; JamalMomeni@eurofins.dk (J.M.); bioinformatics@ron.dk (R.O.N.); RIVIN@vikinggenetics.com (R.V.); 6 Department of Agroecology, Aarhus University, 4200 Slagelse, Denmark; per.kryger@agro.au.dk; 7 Lab of Agricultural Zoology and Entomology, Agricultural University of Athens, 11855 Athens, Greece; mbouga@aua.gr; 8 LLH Bee Institute Kirchhain, 35274 Kirchhain, Germany; marina.meixner@llh.hessen.de

**Keywords:** *Apis mellifera*, population structure, diversity, whole-genome sequencing, pool-sequencing

## Abstract

Background: Whole-genome sequencing has become routine for population genetic studies. Sequencing of individuals provides maximal data but is rather expensive and fewer samples can be studied. In contrast, sequencing a pool of samples (pool-seq) can provide sufficient data, while presenting less of an economic challenge. Few studies have compared the two approaches to infer population genetic structure and diversity in real datasets. Here, we apply individual sequencing (ind-seq) and pool-seq to the study of Western honey bees (*Apis mellifera*). Methods: We collected honey bee workers that belonged to 14 populations, including 13 subspecies, totaling 1347 colonies, who were individually (139 individuals) and pool-sequenced (14 pools). We compared allele frequencies, genetic diversity estimates, and population structure as inferred by the two approaches. Results: Pool-seq and ind-seq revealed near identical population structure and genetic diversities, albeit at different costs. While pool-seq provides genome-wide polymorphism data at considerably lower costs, ind-seq can provide additional information, including the identification of population substructures, hybridization, or individual outliers. Conclusions: If costs are not the limiting factor, we recommend using ind-seq, as population genetic structure can be inferred similarly well, with the advantage gained from individual genetic information. Not least, it also significantly reduces the effort required for the collection of numerous samples and their further processing in the laboratory.

## 1. Introduction

Studying population genetic structure and diversity is the basis of our understanding of biodiversity and the conservation of species [1]. Due to the recent rapid developments in sequencing technology, it is now possible to gain insights into the genomic structure of populations with unprecedented power and accuracy [2]. To make the best use of limited resources, different sampling and sequencing approaches to study population structure are being adopted, which can be summarized as (i) covering whole genomes, but sampling few individuals (e.g., Ref. [3]), (ii) sampling many individuals, but covering limited parts of the genome (e.g., restriction-site associated DNA (RAD) sequencing, exome capture, or genotype-by-sequencing (GBS)) [4,5] or (iii) whole-genome sequencing and pooling of many individuals (pool-seq) (e.g., Ref. [6]). While techniques relying on the reduced representation of the genome are mostly being used in non-model organisms (extensively reviewed, e.g., in [7,8]), the pooled sequence method has been advocated as an alternative, cost-effective approach identifying genome-wide patterns of genetic variation from large populations [9,10].

Allele frequencies are one of the key parameters to study population genetic structure and to estimate genetic distances between populations [1], and the power of many genetic analyses increases with the accuracy of the allele frequency estimates derived from population samples [10]. When sequencing individually a limited number of samples per population, the estimate of the allele frequencies and the sampling variance stems directly from the selection of individuals [11]. By pool-sequencing, on the contrary, numerous samples from a given population can be analyzed, notably reducing the sampling error. Thus, pool-seq has been statistically shown to produce more accurate estimates of population allele frequencies at a lower cost than sequencing of individuals [9,11,12,13].

The cost-effectiveness of pool-seq becomes obvious when considering the cost of individual handling and library preparation for sequencing: as one pool represents a single sample, only one library needs to be prepared [6,10]. As sequencing costs continue to decrease, library preparation is becoming an increasing factor to consider within the research budget.

Pool-seq is especially suitable for applications that require large sample sizes and the analysis of multiple samples/populations; however, for an optimal design of pools, previous knowledge of population structure is beneficial or even necessary. On the other hand, the disadvantage of pool-sequencing is the loss of genetic information at the individual level. Therefore, this technique may not be suitable for certain applications [10,11], for instance, in cases where population boundaries are not clear or gradual or after recent contact with populations. In such cases, individual whole-genome sequencing is better suited, but may not be feasible for several hundreds to thousands of individuals [14].

Most of the advantages and disadvantages of the pool and individual sequencing approaches have been discussed theoretically or by using simulation studies [6,10,11,15]. Thus, it is not entirely clear when, in practice, pool-seq or ind-seq would be the method of choice for a given population genetic study, and actual case studies are needed to reach an empirical consensus on the circumstances in which one approach is better suited over the other.

To directly compare the pool-seq and ind-seq approaches and evaluate their ability to infer population structure and diversity, we apply both methods to the same populations of European honey bees (*A. mellifera* L.). Both approaches, whole-genome ind-seq and pool-seq, have been previously applied in this species, albeit not with a comparative purpose (e.g., [16,17]). Europe, with its numerous autochthonous honey bee subspecies belonging to four different evolutionary lineages [18,19,20,21,22,23], holds a large fraction of the *A. mellifera* genetic heritage. Substantial geographic variation in European honey bee populations has been investigated and described in several previous studies [24,25,26,27,28,29]. Nonetheless, while the genetic variation of honey bees in some parts of Europe has been subject to detailed studies and a considerable level of knowledge has been accumulated ([29,30,31,32,33,34,35] amongst others), some other areas, especially in the eastern part of the continent, have received comparatively little scientific interest [36,37]. Consequently, the analysis and description of honey bee subspecific variation in Europe cannot be regarded as complete, and the taxonomic status of some populations is not yet fully resolved. Thus, comparatively little information is available regarding a global analysis of the genetic variability of *A. mellifera* in Europe. Moreover, because of modern apicultural management and activities, such as queen trade or migratory beekeeping, the distribution and genetic diversity of European honey bees in many places no longer correspond to their natural state [32,38].

In this study, we empirically assess the consistency of two sequencing approaches, pool-seq and ind-seq, in estimating allele frequency, genetic diversity and documenting population structure in European *A. mellifera* honey bees. To this end, we sampled a total of 1347 worker bees from 14 populations, covering a large range of *A. mellifera* subspecies and diversity in Europe, and with a special focus on areas where studies had been scarce, especially towards the eastern range limit of the species. We sequenced the whole-genome of pools from about 90 workers (pool-seq), as well as ten individuals per population (ind-seq). Data obtained by either pool-seq and ind-seq were then compared to their ability to detect the population structure and diversity of European honey bees. Finally, we discuss the advantages and disadvantages of both approaches in practice and recommend future studies.

## 2. Materials and Methods

### 2.1. Sampling

**Figure 1:**
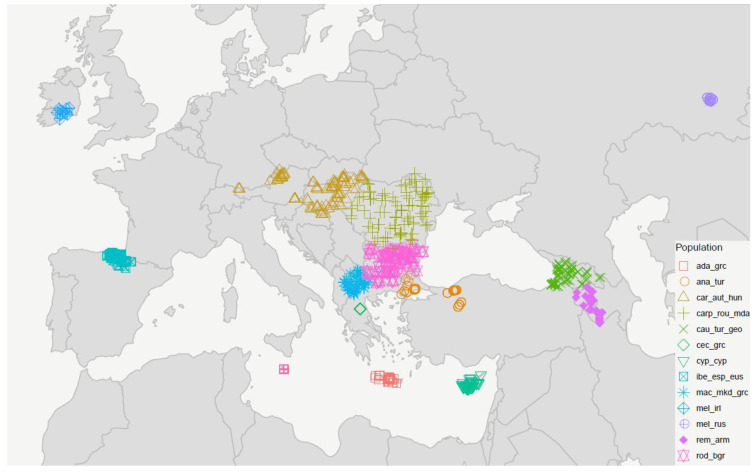
Sampling locations in Europe and adjacent regions plotted in R using ggplot2. M-lineage: ibe_esp_eus = *A. m. iberiensis* from Spain; mel_irl = *A. m. mellifera* from Ireland; mel_rus = *A. m. mellifera* from Russia; C-lineage: car_aut_hun = *A. m. carnica* from Austria and Hungary; rod_bgr = *A. m. rodopica* from Bulgaria; carp_rou_mda = *A. m. carpatica* from Romania and Moldova; mac_mkd_grc = *A. m. macedonica* from North Macedonia a Northern Greece; cec_grc = *A. m. cecropia* from Greece; ada_grc = *A. m. adami* from Crete, Greece; O-lineage: cyp_cyp = *A. m. cypria* from Cyprus; ana_tur = *A. m. anatoliaca* from Turkey; rem_arm = *A. m. remipes* from Armenia; cau_tur_geo = *A. m. caucasia* from North-East Turkey and Georgia; and A-lineage: rut_mlt = *A. m. ruttneri* from Malta. Locations of mel_irl samples are exemplary, as exact coordinates are unavailable.

A sampling strategy was devised to collect representative samples of worker bees from the entire range of *A. mellifera* in Europe (Figure 1, Table 1). Areas previously poorly studied, especially towards the eastern range limit of the species, were given special consideration. In total, we sampled 14 populations, each one represented by 80–100 worker bees from unrelated colonies (one worker bee per apiary). Following the *A. mellifera* subspecies nomenclature by Engel et al. [39], with some deviations (as in Momeni et al. [17]), the sampled populations in this study belong to four evolutionary lineages and 13 different subspecies: Lineage M: *A. m. mellifera* Linnaeus 1758, and *A. m. iberiensis* Engel 1999; Lineage A: *A. m. ruttneri* Sheppard et al. 1997 [20]; Lineage O: *A. m. anatoliaca* Maa 1953, *A. m. caucasia* Pollmann 1889, *A. m. remipes* Gerstaecker 1862, and *A. m. cypria* Pollman 1879; Lineage C: *A. m. cecropia* Kiesenwetter 1860, *A. m. carnica* Pollman 1879, *A. m. macedonica* Ruttner 1988, *A. m. adami* Ruttner 1975, *A. m. carpatica* Foti 1965 [40], and *A. m. rodopica* Petrov 1991 [41]. Based on mitochondrial DNA, *A. m. caucasia* has also been assigned to the C lineage [42]. In this study, we refer to the 14 populations based on the subspecies and country abbreviations, as presented in Table 1.

### 2.2. DNA Extraction and Sequencing

For this study, we pooled about 90 individuals per population following Schlötterer et al. [10], who recommended a sampling size of 40 to 100 individuals. To assemble one pool for sequencing, the heads without eyes of up to 100 workers were ground together, and the DNA was extracted following standard methods [45]. Sequencing libraries of each pool-DNA were constructed with the TruSeq DNA PCR-Free library preparation kit and sequenced on an Illumina HiSeq 2500 platform (Illumina Inc., San Diego, CA, USA)) one lane per pool.

DNA from the thorax of ten workers (each one from a different colony), except for *A. m. adami* with only nine workers available, from each of the 14 pools, were extracted individually using the CTAB method [46]. Sequencing libraries were generated using NEB Next^®^ Ultra DNA Library Prep Kit for Illumina^®^ (New England Biolabs Inc., Ipswich, MA, USA) following the manufacturer’s recommendations. Libraries were sequenced on the Illumina X Ten platform (Illumina Inc., San Diego, CA, USA). The very same samples sequenced individually have also been included in the respective pools.

For the two approaches, two different types of DNA extractions were used from different parts of the bodies (head, thorax), followed by different library preparation protocols, and finally, two different sequencing platforms were used. This strategy was chosen in order to compare the two methods in very realistic settings, that is, we used the same initial populations, then applied the two approaches independently, including the sequencing at different facilities, and bioinformatics analyses by different researchers, such that finding good agreement between the two approaches would indicate robustness to the method used.

### 2.3. Sequencing Data Processing

Bioinformatics processing of the generated pool sequence data was performed using best practices following Schlötterer et al. [10]. Illumina adaptors and low-quality bases were removed using Trimmomatic v0.32 [47], and read quality was checked with FastQC (http://broadinstitute.github.io/fastqc, accessed on 3 November 2019). High-quality sequences were mapped against the honey bee reference genome Amel4.5 [48] using bwa-mem 0.7.10 [49]. SAMtools v0.1.19 [50] and Picard-tools v1.124 (http://broadinstitute.github.io/picard/, accessed on 3 November 2019) were used to convert between SAM and BAM formats, remove duplicate reads, sort the BAM files, remove reads with low-quality mapping (MAPQ < 20), and keep only properly mapped pairs. Subsequently, the data were processed following the steps of the PoPoolation package [51]. The mapping files were split by chromosome to speed up the analyses, converted to mpileup, and indels were removed. Finally, using a minimum count of 3, the data for the different pools were subsampled to uniform coverage (50×) to allow comparability between under-sequenced and over-sequenced pools. The entire strategy for pooled sequence analysis described here was successfully applied in previous studies [17,52], and is available as an automatized Snakemake pipeline [53,54] at https://github.com/jlanga/smsk_popoolation (accessed on 3 November 2019).

For individual sequencing data, reads were filtered by fastp [55] to exclude those with excessive low-quality bases. Clean reads were mapped to the Amel4.5 reference genome [48] using the bwa-mem aligned [56]. Variants were called using SpeedSeq pipeline [57] and default settings. We removed indels, SNPs within ten bp of indels, and SNPs within the repeat regions. The remaining SNPs were further filtered, and only high-quality ones meeting the following criteria were kept: (1) biallelic; (2) quality score > 30; (3) missing genotype <10%.

### 2.4. Allele Frequency Correlation

To compare the genetic variability identified based on the two different sequencing approaches, the allele frequencies of the commonly called SNPs in each population were calculated using PLINK1.9 [58] for ind-seq and a custom-built Python script for pool-seq. For each population, the allele frequencies estimated by both approaches were correlated with Pearson’s correlation coefficient [59] using the R [60] package ggpubr [61] and plotted using ggplot2 [62].

### 2.5. Genetic Diversity between and within Populations

Genetic diversity between populations was inferred using F_ST_ distances [63]. For pools, we used PoPoolation2 [51] to calculate pairwise F_ST_ in overlapping window sizes of 20 kb and 10 kb step-size. For ind-seq data, we first used ANGSD [64] to estimate genotype likelihoods from the mapped reads, with loci from repeated regions, low mapping quality (minMapQ < 30), or low base quality (minQ < 20) removed. In addition, we only kept loci covered in at least 100 individuals. Based on the probabilities, we then used the realSFS function to calculate F_ST_ between pairs of populations, using a sliding windows approach (window size 20 kb, step size 10 kb).

Expected heterozygosity (He), as a measure of genetic diversity within populations, was calculated for each population with a custom R script using the above-estimated allele frequencies by pool-seq and ind-seq and the standard formula (2pq) [63].

Genetic diversity and F_ST_ results were plotted in R [60] using the ggplot2 package [62]. Pearson’s correlation coefficients [59] were calculated between heterozygosity and pairwise F_ST_ estimates as calculated by the pool-seq and ind-seq approaches, respectively, using the ggpubr package in R [61].

### 2.6. Population Structure

To infer population structure with the pool-seq data, a principal component analysis was performed using the allele frequencies for each population. For ind-seq data, we first used ANGSD [64] to estimate genotype likelihoods as described above, but with an additional filter to keep loci with a minor allele frequency of no less than 0.05. Based on the posterior genotype probability, PCAngsd [65] was used to calculate the covariance matrix, and ngsDist [66] was used to calculate pairwise genetic distances.

For ind-seq data, population structure was further investigated using a model-based approach. NGSadmix [67] was used to estimate admixture proportions from K = 2 to K = 20, with ten runs for each K. Optimum numbers of K clusters were determined using DeltaK on CLUMPAK [68,69].

Principal components (PCs) and individual model-based ancestries were plotted in R [60] with the ggplot2 package [62].

## 3. Results

### 3.1. Sequence Data and Variants

For the pool sequencing approach, we obtained 4,372,477,988 raw reads in total, resulting in an overall genome depth of coverage >1800×. Mean coverage for each pool ranged from 70.3× (cec_grc) to 264.5× (rod_bgr) (Table S1). For the individual sequencing approach, a total of 4,060,632,652 raw reads were generated, resulting in an overall genome depth of coverage ~2600×, with a mean coverage of 17.7× (Table S2).

### 3.2. Allele Frequency Correlation

**Figure 2:**
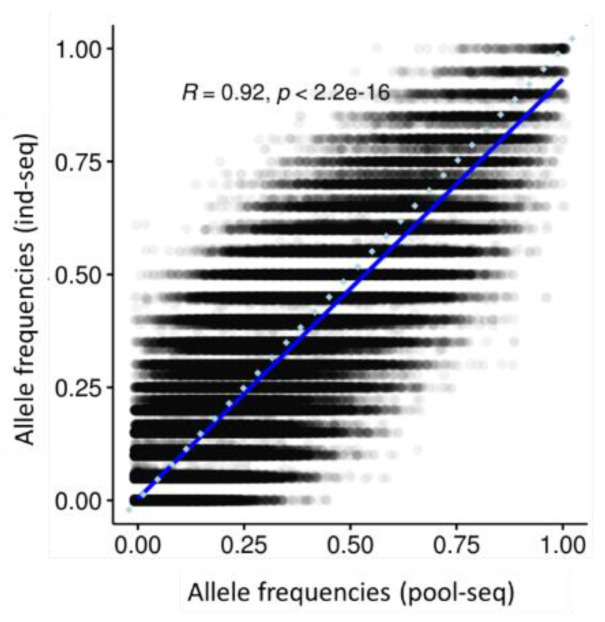
Allele frequency correlation between pool- and ind-seq in each population.

A total of 1.6 M and 3.7 M SNPs were called for pool- and ind-seq, respectively. Variants called by both approaches were extracted, leaving 607 K SNPs to calculate the correlations between the allele frequencies generated with either method in each population. The allele frequencies generated with the two methods were very highly correlated with each other, but high variation was observed for single variants (Figure 2; R = 0.92, *p* > 0.001).

### 3.3. Genetic Diversity between and within Populations

**Figure 3:**
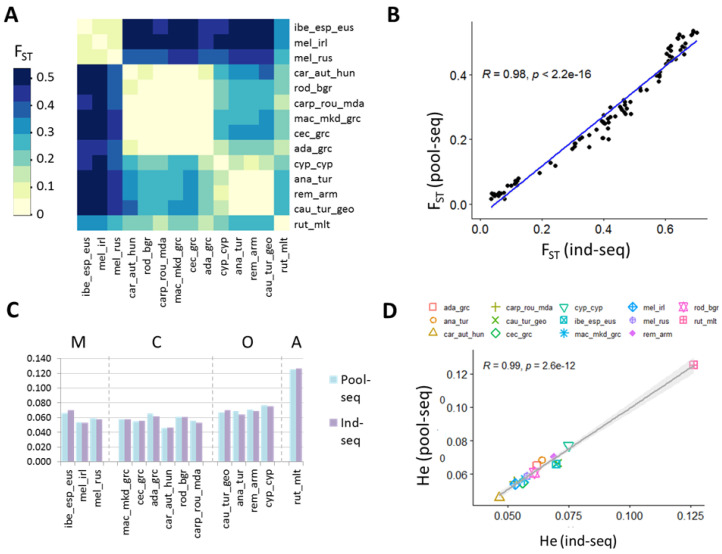
Genetic variation between and within populations. (**A**) Heatmap based on whole-genome pairwise average FST between each population pair calculated from pool-seq data. (**B**) Correlation of pairwise FST estimates between pool-seq and ind-seq data. (**C**) Expected heterozygosity for each population as estimated by pool and ind-seq. (**D**) Correlation between heterozygosities as estimated by pool-seq and ind-seq data. M-lineage: ibe_esp_eus = *A. m. iberiensis* from Spain; mel_irl = *A. m. mellifera* from Ireland; mel_rus = *A. m. mellifera* from Russia; C-lineage: car_aut_hun = *A. m. carnica* from Austria and Hungary; rod_bgr = *A. m. rodopica* from Bulgaria; carp_rou_mda = *A. m. carpatica* from Romania and Moldova; mac_mkd_grc = *A. m. macedonica* from North Macedonia a Northern Greece; cec_grc = *A. m. cecropia* from Greece; ada_grc = *A. m. adami* from Crete, Greece; O-lineage: cyp_cyp = *A. m. cypria* from Cyprus; ana_tur = *A. m. anatoliaca* from Turkey; rem_arm = *A. m. remipes* from Armenia; cau_tur_geo = *A. m. caucasia* from North-East Turkey and Georgia; and A-lineage: rut_mlt = *A. m. ruttneri* from Malta.

The analysis of genetic diversity between populations revealed two hierarchical levels of differentiation that were consistently observed based on both pooled populations and individual sequence data: High divergence between populations of different evolutionary lineages (average F_ST Pools_ = 0.41 ± 0.11 SD; F_ST Ind_ = 0.51 ± 0.12 SD; Table S3) and low divergence between populations of the same lineage (average F_ST Pools_ = 0.06 ± 0.03 SD; F_ST Ind_ = 0.09 ± 0.06 SD) (Figure 3A, Tables S4 and S5). While F_ST_ estimates inferred from ind-seq data (F_ST Ind_ = 0.40 ± 0.22 SD; Table S5) were higher in all pairwise comparisons than the ones calculated using pool-seq data (F_ST Pools_ = 0.27 ± 0.17 SD; Table S4), the correlation between both approaches is extremely high (Figure 3D, R = 0.98, *p* < 0.001), and its visualization as a distance heatmap revealed near identical results (Figure 3B).

Similarly to the allele frequencies and genetic distances, expected heterozygosities were very highly correlated between pool- and ind-seq (Figure 3D; R = 0.98, *p* = 0.037), and revealed nearly identical results of diversity within populations as estimated by both approaches (Figure 3C): The highest genetic diversity by far was identified in the rut_mlt population which belongs to the African evolutionary lineage. Followed by O lineage populations which have a significantly higher mean diversity (He_Pools_ = 0.071, He_Ind_ = 0.070) than the mean C lineage (He_Pools_ = 0.057, He_Ind_ = 0.056, *p* = 0.002) and mean M lineage (He_Pools_ = 0.059, He_Ind_ = 0.060, *p* = 0.031) diversity. The lowest diversity overall, as estimated by pool-seq and ind-seq, was found in the car_aut_hun pool from Austria and Hungary belonging to the C-lineage.

### 3.4. Population Structure

**Figure 4:**
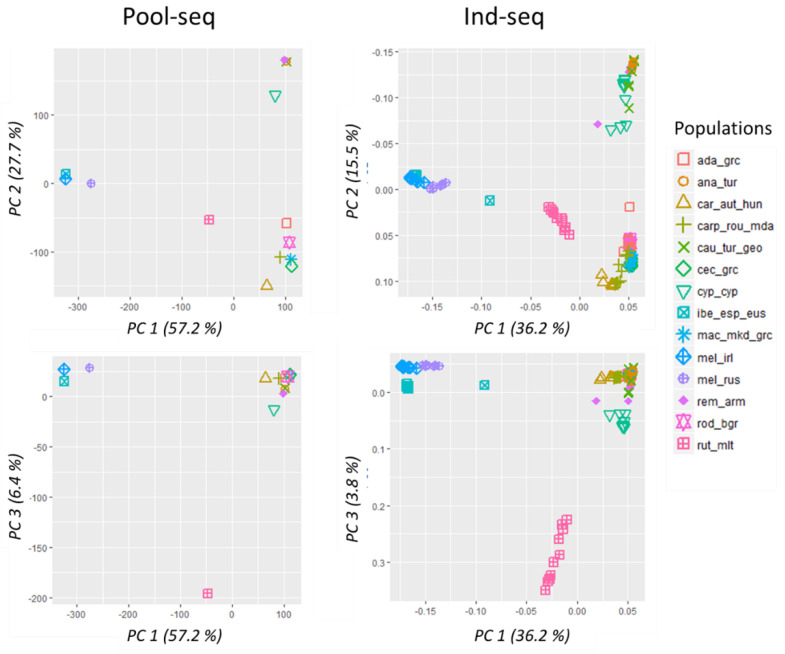
Principal component analysis (PCA) of pool-seq data (**left two panes**) and ind-seq data (**right panes**). Upper panels show the first and second principal components explaining most of the variance, while the lower panels display the first and third components, which only account for 6.4% and 3.8% of the total variation, respectively. M-lineage: ibe_esp_eus = *A. m. iberiensis* from Spain; mel_irl = *A. m. mellifera* from Ireland; mel_rus = *A. m. mellifera* from Russia; C-lineage: car_aut_hun = *A. m. carnica* from Austria and Hungary; rod_bgr = *A. m. rodopica* from Bulgaria; carp_rou_mda = *A. m. carpatica* from Romania and Moldova; mac_mkd_grc = *A. m. macedonica* from North Macedonia a Northern Greece; cec_grc = *A. m. cecropia* from Greece; ada_grc = *A. m. adami* from Crete, Greece; O-lineage: cyp_cyp = *A. m. cypria* from Cyprus; ana_tur = *A. m. anatoliaca* from Turkey; rem_arm = *A. m. remipes* from Armenia; cau_tur_geo = *A. m. caucasia* from North-East Turkey and Georgia; and A-lineage: rut_mlt = *A. m. ruttneri* from Malta.

Overall, the population structure inferred by principal component analysis (PCA) based on data generated with both sequencing approaches was nearly identical, showing a clear separation of the populations into the four main lineages (Figure 4): The first component (PC1) separates the M-lineage from the O and C-lineage, that in turn are separated by the second component (PC2) (Figure 4). In these PCA plots, rut_mlt samples are placed close to the center, and they are clearly distinguished from the rest of the samples on PC3 (Figure 4). PCA by ind-seq further identifies two outliers (one individual each of the ibe_esp_eus and rem_arm population) that are placed distantly to their group members.

**Figure 5:**
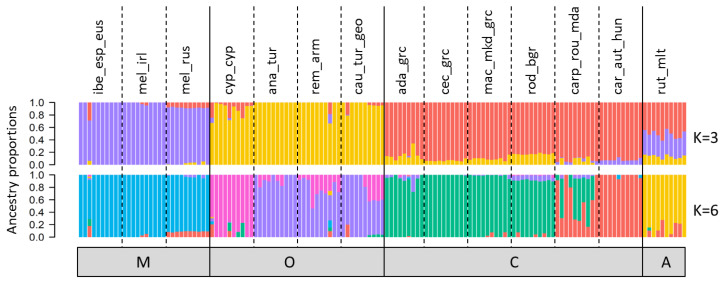
Model-based ancestry as calculated with NGSAdmix for best (K = 3) and second-best (K = 6) number of K ancestral populations. Each individual is represented by a vertical bar and colored according to the proportion of the genome that was derived from one of K clusters. Samples are ordered according to evolutionary lineage and sampling population. M-lineage: ibe_esp_eus = *A. m. iberiensis* from Spain; mel_irl = *A. m. mellifera* from Ireland; mel_rus = *A. m. mellifera* from Russia; C-lineage: car_aut_hun = *A. m. carnica* from Austria and Hungary; rod_bgr = *A. m. rodopica* from Bulgaria; carp_rou_mda = *A. m. carpatica* from Romania and Moldova; mac_mkd_grc = *A. m. macedonica* from North Macedonia a Northern Greece; cec_grc = *A. m. cecropia* from Greece; ada_grc = *A. m. adami* from Crete, Greece; O-lineage: cyp_cyp = *A. m. cypria* from Cyprus; ana_tur = *A. m. anatoliaca* from Turkey; rem_arm = *A. m. remipes* from Armenia; cau_tur_geo = *A. m. caucasia* from North-East Turkey and Georgia; and A-lineage: rut_mlt = *A. m. ruttneri* from Malta.

Model-based ancestry was further investigated in individual samples. The optimal number of clusters as inferred by Evanno’s DeltaK was K = 3 (Figure S1) that separated the individuals into three major clusters coinciding with the lineages M, O, and C, and leaving rut_mlt individuals with an intermediate mixed genetic background (Figure 5, top panel). The second-best K = 6 (Figure S2) separates rut_mlt into its own cluster and, within the O-lineage, differentiates cyp_cyp from the other three subspecies. Also, within this lineage, a substructure within cau_tur_geo becomes visible, where about half the samples display two different ancestries. At K = 6, we further observe a differentiation within the C-lineage, between car_aut_hun from the northern part of the distribution and all other populations (mac_mkd_grc, cec_grc, ada_grc, rod_bgr) in the southern part of the lineage range; however, the individuals of carp_rou_mda display mixed ancestry with varying degrees of both genetic backgrounds. Individuals with a mixed genetic background were also identified in a few other populations, for instance, one each of ibe_esp_eus and rem_arm has already seen in the PCA, but also several rut_mlt and cyp_cyp individuals.

## 4. Discussion

Whole-genome sequencing of individuals provides genetic data at the highest resolution. However, this rather expensive approach can only be applied to a limited sample size, while pool-seq grants data for a much larger sample set. In this study, we empirically evaluated these approaches to infer population structure and diversity in a real dataset of European *A. mellifera* populations. Comparing the two different sequencing approaches (pool-seq and ind-seq), we found that both revealed a population genetic structure and genetic diversities of European honey bees that were nearly identical. Moreover, the results are in good concordance with previous findings (e.g., [16,18,19,70]), although most previous studies were based on different populations and smaller datasets. As either method comes with specific advantages, cost-effectiveness in the case of pool-seq and depth of information in the case of ind-seq, a cost-efficient strategy could be to combine both approaches.

### 4.1. The Limited Ability of Pool-Seq to Identify Low-Frequency Variants

The pool-seq approach also identified only a fraction of the SNPs found by ind-seq. However, this difference is expected, considering that the total sequencing depth of individuals (2600× for 139 individuals) was higher than the one for pools (1800× for 14 pools of 100 individuals each). In addition, by subsampling pool sequence data to uniform coverage and requiring a strict filter of minimum read count 3 to call a variant, we could only detect SNPs with a minor allele frequency of >0.06. This was already pointed out by Cutler and Jensen [11], who showed that low-frequency variants are lost in pool-seq experiments when appropriate call filters are set that consider sequencing error rates. Moreover, variants may remain undetected if equal molar concentrations represent not all individuals in the pool. While we cannot rule out this possibility in our experiments, any variance because of pooling is expected to be small, as about 90 individuals were used in each pool. In any case, the inference of population structure is not influenced by the exact number of variants. On the contrary, rare and low-frequency variants are typically filtered for such analyses (e.g., Refs. [6,71,72]). Also, the importance rather lies in the accurate estimates of common variants [10]. However, for other applications such as the association of rare variants to disease or specific phenotypes [73,74], this point might be more critical and needs to be considered in the sampling and sequencing strategy, as advised elsewhere [10,11].

### 4.2. Sampling and the Importance of Previous Knowledge for Pool-Seq

The total sample size of individuals that are pooled is a crucial parameter that influences the accuracy of the allele frequency estimations [75,76]. By performing a large-scale and comprehensive sampling and including ~90 individuals per pool, we ensured that the allele frequencies obtained by pool-seq could be regarded as representative of the populations of the subspecies and regions under study. In contrast, allele frequency estimation based on ind-seq relies on the few samples chosen per population, ten in our case. Therefore, pooling is advantageous if it can be based on prior knowledge of the population structure. It is thus important to include additional data, for instance from morphology or previous genetic studies, when deciding which individuals should be included in a pool. Although we were able to base the sampling in some of our populations on previous genetic studies (see references in [17]), in some populations admixed individuals were identified (e.g., rem_arm, cau_tur_geo, carp_rou_mda, rut_mlt). Here, pool-based estimates could potentially yield biased information, as the presence of hidden substructures will obscure the estimated mean allele frequencies. Moreover, any possibly present Wahlund effect [77] caused by hidden substructures would not be detectable either, since the observed heterozygosity is not accessible via pool-seq. In other words, pooling limits us to the overall view by assuming genetic homogeneity between the individuals that constitute the population sample, so any potentially present heterogeneity within the population cannot be detected. In consequence, it is recommendable to use caution in choosing the individuals for a sequencing pool and, if in doubt about genetic homogeneity, to exclude individuals or to set up separate pools.

### 4.3. Near Identical Inference of Population Structure and Diversity by Pool-Seq and Ind-Seq

A remarkable finding in our study is that, despite the huge difference in sampling coverage between pool-seq and ind-seq, allele frequencies obtained with the two methods correlate extremely well with each other (R = 0.92, Figure 2), indicating that with as few as 10 individuals, good estimates of average population structure can be achieved. This is further evidenced by the correlation of F_ST_ values (Figure 3B) which reveals near identical results between the two approaches. Equally highly correlated were the genetic diversities within populations as estimated by expected heterozygosity (Figure 3D). Similar to our results, Dorant et al. [6] evaluated genotyping-by-sequencing, pool-seq, and RAD capture approaches and identified very congruent results by the three tested methods in identifying weak population structure of a Homarus americanus population (correlation coefficients R > 0.9). Also, in natural populations of *Arabidopsis halleri*, a non-model plant species, highly correlated allele frequencies (R > 0.98) were identified between pool-seq and ind-seq. Thus, although only few studies empirically evaluated the accuracy of allele frequency estimates derived from pool-seq and ind-seq in natural populations, all arrive at very high correlations and demonstrate that either approach applies to population genomics studies.

### 4.4. Both Approaches Compare Well to Established Studies

Regarding the population structure and differentiation, it is quite remarkable that our results based on using whole-genome sequence data at a high depth and of a comprehensive and unprecedented sample set are comparable to the population genetic structure of European honey bees proposed by F. Ruttner in the 1980s based on morphometric analyses [18]. Namely, high genetic divergences are found between the four lineages, while moderate and minor differences appear between subspecies within lineages. This finding is consistent with other published literature throughout the years studying different populations and using different tools from classical morphometry [18,30,36], over geometric-morphometrics [37], to microsatellites [27,31,35] and SNP markers [16,17]. Hence, while advanced technologies allow us to sequence samples at the whole-genome level and thereby gain maximal data, if the aim is simply to only infer population structure, whole-genome sequencing can be an overshoot, in particular in conservation genomics applications [1,78]. In contrast, if the aim is to identify local adaptations in natural populations, whole-genome sequencing enables the identification of signatures of selection [79,80,81,82], a limited number of genetic markers cannot identify that. For this application, pool-seq has also been successfully applied before [52,83,84,85].

The highest genetic diversity by far was identified in the rut_mlt population as a representative of the African evolutionary lineage (Figure 3A), which is known to be the lineage with the highest genetic diversity as identified in previous studies [16,72,86]. The lowest diversity was found in the car_aut_hun population from Austria and Hungary belonging to the C-lineage (Figure 3A). Already in other studies *A. m. carnica* has been identified as the subspecies with the lowest genetic diversity [16,38]. A possible explanation of the lower diversity consistently identified in this subspecies could be genetic drift caused by selective breeding, as *A. m. carnica* is one of the most popular honey bee subspecies used for breeding [87]. Among the O lineage, the cyp_cyp population, despite originating from a small island, revealed the highest expected heterozygosity in this group, most likely due to hybridization with other subspecies that are imported to the island, and known to increase diversity [86,88]. Moreover, within the M lineage the highest genetic diversity was identified in the ibe_eus_esp population, in concordance with the Iberian Peninsula being described as a glacial refuge of M-lineage diversity [89,90], while the two *A. m. mellifera* populations reflect distant (mel_rus) or isolated (mel_irl) populations, which may thus potentially have lost diversity through genetic drift during recolonization. In particular, in the case of the *A. m. mellifera* island population of Ireland, where limited human inference through importation, is suspected [43,91].

### 4.5. The Cost-Benefit Ratio between Pool-Seq and Ind-Seq

**Figure 6:**
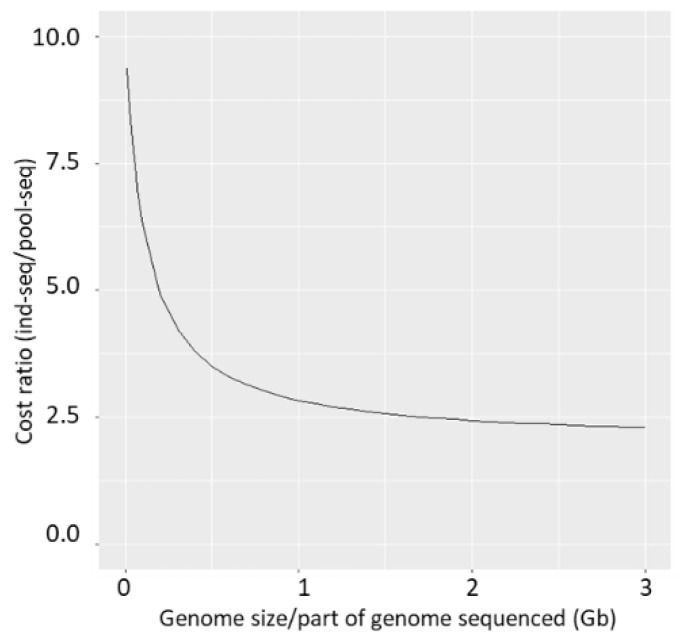
Cost ratio ind-seq/pool-seq. The x-axis represents the genome size or part of the genome sequenced (Gb) in case of reduced representation techniques. The y-axis is the ratio of the total cost of ind-seq/pool-seq (assuming 1400× total coverage for ind-seq and 700× total coverage for pool-seq).

Since similar results are obtained with both sequencing approaches and results are concordant with published literature, the issue of costs involved with either approach gains additional weight. In general, the cost of pool-seq is considered lower [9,12,13]. To enable a direct comparison, we recently (2021) enquired sequencing quotes similar to the magnitude of our study with pool-seq (14 pools, 14 extractions, 14 library preparations, and total target depth 1400×) and ind-seq (140 individuals, 140 extractions, 140 library preparations, total depth 1400×) from a European and from a Chinese company. For ind-seq the estimates were 19,689 € and 44,800 ¥, respectively, while for pool-seq they were 7003 € and 19,600 ¥, that is, 65% to 55% less expensive. This considerable price difference between the two approaches is certainly a relevant point of consideration for scientists with low to medium research budgets. It is to note that for organisms with relatively small genome size, such as the honey bee (236 Mb), the extraction and library preparation steps constitute a large portion of the costs in comparison with the cost of actual sequencing, and therefore, applying the pool-seq approach becomes more cost-efficient. In contrast, when studying species with large genomes, such as plants (e.g., Ref. [15]), the cost of the actual amount of sequence data (Gb) needed will account for a higher proportion of the total cost and increase for both methods, resulting in a smaller difference between them (Figure 6). On the other hand, for reduced representation approaches such as RAD-seq, GBS, or exome capture, for which the actual sequenced part of the genome can be very small, pool-seq will become much more affordable, e.g., the cost ratio approaching 10, as the sequencing cost would be very low (Figure 6), highlighting the advantages of pool-seq for ecological studies of non-model organisms [6,92,93].

On the other hand, besides the cost of sequencing itself, we need to consider the actual cost of collecting the samples required for either approach. In our case, the sampling of ~90 individuals per population, each one originating from a different colony and apiary and a total of 16 different countries, constituted a huge effort and generated extra costs. In comparison, the sampling of only ten individuals per population is less complex and labor-intensive.

### 4.6. Additional Insights from Ind-Seq

While pool-seq and ind-seq reveal similar overall results on population structure and genetic diversity, albeit, at different costs, ind-seq can provide additional information at the individual level. In this way, we could identify individuals with a mixed genetic background that is indicative of the existence of hybridization between subspecies and that would give rise to a greater resemblance between populations when analyzed by pooling. For instance, by natural hybridizations in the contact areas of two subspecies, as we find between *A. m. remipes* and *A. m. caucasia* on the borders between Turkey and Armenia, and between lineages as has been reported previously [29,89,94,95,96]. Or hybridizations due to contemporary human-mediated processes, as we see in the case of *A. m. ruttneri* on the island of Malta [97].

An additional advantage of individual whole-genome sequence data is that further analyses can be performed, such as the inference of evolutionary and demographic histories [72,98,99,100]. Although not the focus of this study, such analyses can give additional insights that may be particularly important for non-model species and in a conservation context [1,101].

## 5. Conclusions

Our case study on *A. mellifera* is a good example to evaluate two methods. The species is well studied, and we could compare our results with previous studies that used different tools and populations. While overall results of both approaches were very similar, our final verdict for the empirical comparison between pool-seq and ind-seq to investigate population structure in the honey bee is that ind-seq, albeit more expensive, could give us similar or even equal information, while additionally providing insights into individual-based admixture. Based on our experience, we would thus not necessarily recommend to only applying the pool-seq approach in similar studies. Nevertheless, pool-seq can be a useful and cost-efficient option, for instance for variant discovery [17,102], and/or in combination with ind-seq for other less well-studied species.

Both approaches, pool-seq and ind-seq, enabled us to get a global vision of European honey bee diversity. The population structure of some of the studied populations was genetically examined for the first time. It will be interesting to analyze them more in-depth to shed light on complex patterns of diversity. For instance, within the C lineage, where multiple highly interrelated subspecies exist in close geographical proximity, we found some level of mixed genetic background in several populations. The comprehensive genomic dataset generated by this study will therefore be the basis for future studies to explore further the genetic variation within and among subspecies and to identify signatures of local adaptations.

## Figures and Tables

**Table 1:** Sample sizes and origin of the 14 populations used in this study.

Lineage	Population	Subspecies	Country	Pool Sequencing Samples (N)	Individual Sequencing Samples (N)	Origin of Samples/References
M	ibe_esp_eus	*A. m. iberiensis*	Spain	100	10	Miguel et al., 2007 [35]
	mel_irl	*A. m. mellifera*	Ireland	100	10	Hassett et al., 2018 [43]
	mel_rus	*A. m. mellifera*	Russia (Ural)	100	10	This study, Momeni et al., 2021 [17]
C	car_aut_hun	*A. m. carnica*	Austria & Hungary	100	10	This study, Momeni et al., 2021 [17]
	rod_bgr	*A. m. rodopica*	Bulgaria	95	10	This study, Momeni et al., 2021 [17]
	carp_rou_mda	*A. m. carpatica*	Romania & Moldova	90	10	This study, Momeni et al., 2021 [17]
	mac_mkd_grc	*A. m. macedonica*	North Macedonia & N-Greece	86	10	This study, Uzunov et al., 2014 [27]
	cec_grc	*A. m. cecropia*	Greece	93	10	This study, Momeni et al., 2021 [17]
	ada_grc	*A. m. adami*	Greece (Crete)	88	9	This study, Momeni et al., 2021 [17]
O	cyp_cyp	*A. m. cypria*	Cyprus	100	10	This study, Momeni et al., 2021 [17]
	ana_tur	*A. m. anatoliaca*	Turkey	100	10	This study, Francis et al., 2014 [44]
	rem_arm	*A. m. remipes*	Armenia	90	10	This study, Momeni et al., 2021 [17]
	cau_tur_geo	*A. m. caucasia*	NE-Turkey & Georgia	105	10	This study, Momeni et al., 2021 [17]
A	rut_mlt	*A. m. ruttneri*	Malta	100	10	This study, Momeni et al., 2021 [17]
TOTAL				1347	139	

## Data Availability

Data from pool sequencing is available on NCBI’s short read archive (SRA) under accession PRJNA666033. Individual sequencing data is deposited into CNGB Sequence Archive (CNSA) of China National GeneBank DataBase (CNGBdb) with accession number CNP0001986. The pipeline for the analysis of the pool sequence data is available at https://github.com/jlanga/smsk_popoolation (accessed on 3 December 2021).

## Supplementary Materials

The following supporting information can be downloaded at: https://www.mdpi.com/article/10.3390/genes13020182/s1, Table S1: mapping statistics pool-seq data, Table S2: mapping statistics individual whole-genome seq data, Table S3: lineage differentiation (F_ST_) based on pool and individual seq data, Table S4: differentiation between populations (F_ST_) based on pool-seq data, Table S5: differentiation between populations (F_ST_) based on individual seq data, Figure S1: delta k plot of Evanno’s test based on NGSadmix analysis for K = 2–20, Figure S2: delta K plot of Evanno’s test based on NGSadmix analysis for K = 3–20.

## Appendix A

Collaborators of the SMARTBEES WP3 DIVERSITY(in alphabetical order):

Eliza Căuia (Institutul de Cercetare Dezvoltare pentru Apicultura SA, Bucharest, Romania), Mary F. Coffey (University of Limerick, Limerick, Ireland), Thomas Galea (Breeds of Origin, Haz-Zebbug, Malta), Miroljub Golubovski (MacBee Association, Skopje, North Macedonia), Karina Grigoryan (Yerevan State University, Yerevan, Armenia), Fani Hatjina (Department of Apiculture, Agricultural Organization ‘DEMETER’, Thessaloniki, Greece), Rustem Ilyasov (Vavilov Institute of General Genetics of Russian Academy of Sciences, Moscow, Russia), Timothea Ioannou (Department of Agriculture, Limassol, Cyprus), Dimosthenis Isaakidis (Beekeeping Center of Crete, Heraklion, Crete, Greece), Evgeniya Ivanova (University of Plovdiv “Paisii Hilendarski”, Plovdiv, Bulgaria), Irakli Janashia (Agricultural University of Georgia, Tbilisi, Georgia), Irfan Kandemir (Ankara University, Ankara, Turkey), Aikaterini Karatasou (Federation of Greek Beekeepers’ Associations, Larissa, Greece), Enikö Sz. Matray (Hungarian Bee Breeders Association, Budapest, Hungary), David Mifsud (Division of Rural Sciences and Food Systems, Institute of Earth Systems, University of Malta, Msida, Malta), Rudolf Moosbeckhofer (Österreichische Agentur für Gesundheit und Ernährungssicherheit GmbH, Wien, Austria), Alexei G. Nikolenko (Institute of Biochemistry and Genetics, Ufa Federal Research Centre of the Russian Academy of Sciences, Ufa, Russia), Alexandros Papachristoforou (Cyprus University of Technology, Limassol, Cyprus), Plamen Petrov (Agricultural University of Plovdiv, Plovdiv, Bulgaria), Aleksandr V. Poskryakov (Institute of Biochemistry and Genetics, Ufa Federal Research Centre of the Russian Academy of Sciences, Ufa, Russia), Aglyam Y. Sharipov (Shulgan-Tash Nature Reserve, Burzyansky District, Russia), Adrian Siceanu (Institutul de Cercetare Dezvoltare pentru Apicultura SA, Bucharest, Romania), Aleksandar Uzunov (Landesbetrieb Landwirtschaft Hessen, Bee Institute Kirchhain, Kirchhain, Germany; Faculty of Agricultural Sciences and Food, University Ss. Cyril and Methodius, Skopje, Republic of Macedonia), Marion Zammit-Mangion (Department of Physiology and Biochemistry, University of Malta, Msida, Malta).
