# Supplementary material for: Population Structure and Diversity in European Honey Bees (Apis mellifera L.)—An Empirical Comparison of Pool and Individual Whole-Genome Sequencing
Source: Genes (Basel). 2022 Jan 21;13(2):182. doi: 10.3390/genes13020182 (PMC8872436; doi:10.3390/genes13020182)
Supplement: Supplementary file 1 [file genes-13-00182-s001.zip › SupplementaryFigures.pdf]

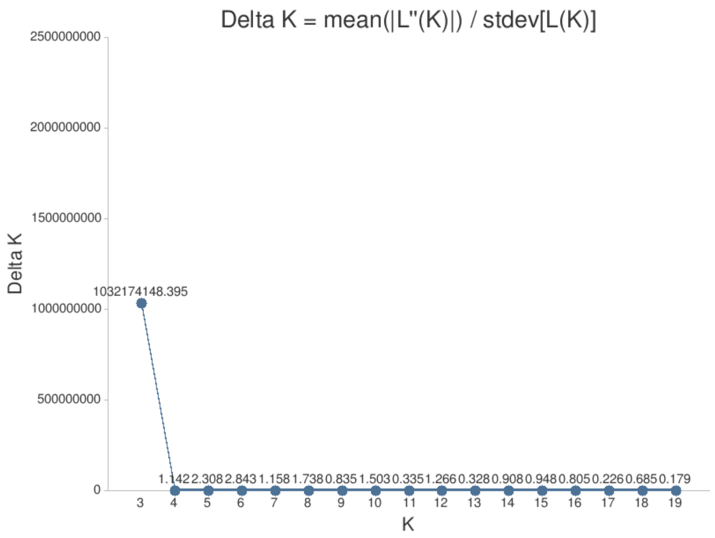

Figure S1. Delta k plot of Evanno's test based on NGSadmix analysis for K = 2 - 20.

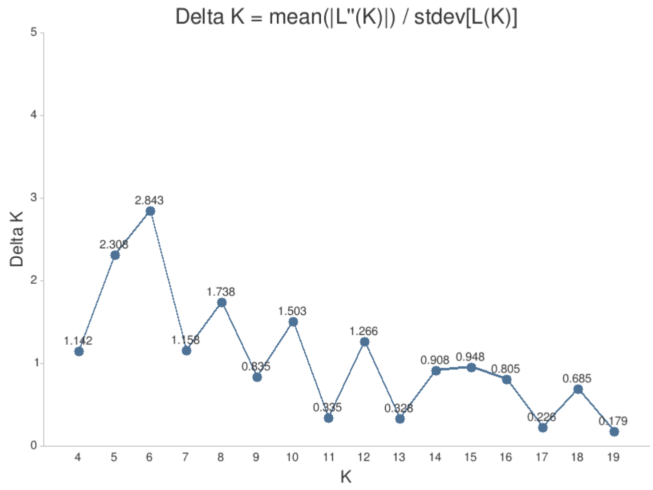

Figure S2. Delta K plot of Evanno's test based on NGSadmix analysis for K = 3 - 20.
